# A Further Exploration of Multi-Slot Based Spectrum Sensing

**DOI:** 10.3390/s19163497

**Published:** 2019-08-09

**Authors:** Jia Zhu, Hongsong Cao, Junsheng Mu

**Affiliations:** 1 College of Mechatronic Engineering, North University of China, Taiyuan 030051, China; 2 School of Information and Communication Engineering, Beijing University of Posts and Telecommunications, Beijing 100876, China

**Keywords:** spectrum sensing, internet of things, cooperative spectrum sensing

## Abstract

Spectrum sensing (SS) exhibits its advantages in the era of Internet of Things (IoT) due to limited spectrum resource and a lower utilization rate of authorized spectrum. In consequence, the performance improvement of SS seems a matter of great significance for the development of wireless communication and IoT. Motivated by this, this paper is devoted to multi-slot based SS in specialty and several important conclusions are drawn. Firstly, SS with one slot outperforms those with multiple slots if decision fusion rule is considered for multi-slot based SS. Secondly, multi-slot based SS is conducive to the performance improvement of SS when instantaneous strong noise occurs in the radio environment. Thirdly, for multi-slot based cooperative spectrum sensing (CSS), majority voting rule among multiple nodes obtains the optimal sensing performance. Both theoretical analysis and simulation experiment validate the conclusions drawn in this paper.

## 1. Introduction

With the rapid development of wireless communication technology and the rapid increasing of wireless communication services, the resources of frequency spectrum are increasingly scarce, even resulting in the increasing crowd of unauthorized ISM (Industrial Scientific Medical) public bands. A recent investigation from Federal Communications Commission (FCC) have shown that the variations in the utilization of the assigned spectrum vary from 15% to 85% [1], which makes the efficient utilization of these bands a more significant problem than the scarcity of the spectrum [2]. In addition, the time scale of spectrum occupancy varies from millisecond to hour [3].

Cognitive radio (CR), with its ability to sense radio environment and exploit the information to opportunistically find unused frequency bands which is best suited for the user’s communication requirements, is viewed as a novel approach to improve spectrum utilization [4]. Cognitive radio consists of three essential components: (1) Spectrum sensing: The secondary users are required to sense and monitor the radio spectrum environment within their operating range to detect the frequency bands that are not occupied by primary users; (2) dynamic spectrum management: Cognitive radio networks are required to dynamically select the best available bands for communication; and (3) adaptive communications: A cognitive radio device can configure its transmission parameters (carrier frequency, bandwidth, transmission power, etc.) to opportunistically make best use of the ever-changing available spectrum.

As the basis of CR, spectrum sensing mainly contributes to the decision on spectrum occupancy in virtue of various signal detection and processing methods. Over the last decade, classical SS schemes contain narrowband sensing and wideband sensing. Narrowband SS analyzes one frequency channel at a time while wideband SS analyzes several frequencies at a time [5]. The main narrowband SS schemes include energy detector (ED) [6], cyclostationary feature detection [7], matched filter detection [8], covariance-based detection [9] and machine learning based sensing [10]. Wideband sensing is mainly devoted to Nyquist based wideband sensing and compressive wideband sensing. ED works as the most popular and classical scheme for spectrum sensing due to its low complexity and superior performance [6]. However, the performance of ED always suffers from noise uncertainty in the radio environment. Even when the background environment is out of the “signal-to-noise radio (SNR) wall”, the capacity of differentiating whether observed spectrum band is busy disappears thoroughly. Consequently, ED with less noise uncertainty deserves to be explored in terms of both performance and reliability improvement for the detection of idle spectrums [11]. 

Generally speaking, there are two parameters closely associated with a spectrum sensing (SS) scheme: Detection probability and false alarm probability. The higher the detection probability is, the better the primary users can be protected. However, from the secondary users’ perspective, the lower the false alarm probability is, the more chances the channel can be reused when it is available, thus the higher the achievable throughput for the secondary users [12]. As a result, the performance of a SS scheme is usually evaluated by the combination of these two factors, such as (1) maximizing detection probability subject to below maximum acceptable false alarm probability, (2) maximizing throughput subject to above minimum tolerable detection probability, (3) minimizing the sum of false alarm probability and probability of miss, etc.

A cognitive user (CU) may have a good line of sight with a primary user, but due to the shadow phenomenon, the existence of the main transmitter (hidden terminal) may not be detected, which is very common in urban/indoor environments. Cooperative spectrum sensing (CSS) solves the hidden terminal issue preferably by placing multiple CR terminals in various locations [13]. With the data gathered by those local terminals or the corresponding judgment results, the observed frequency spectrum is declared to be idle or busy. Although CSS greatly improves the sensing performance, the sensing accuracy is still far below the standard of wireless regional area networks (WRAN) based on CR of 802.22 working group [14]. Motivated by this, multi-slot SS is considered in [15], where the sensing slot in each frame is split into multiple discontinuous mini-slots. The advantages of multi-slot SS in [15] consist in both sides. Firstly, it increases the robustness of the CR system, especially under instantaneous strong noise. For single slot SS, the sensing function of CR may not work due to the high false alarm probability. Secondly, it helps to improve sensing accuracy. The joint decisions of multiple nodes will lower the error probability of idle spectrum detection. However, the work in [15] is mainly devoted to the analysis of data fusion based SS in the multi-slot fashion. When the situation with limited storage or computing power occurs, the work in [15] is not the optimal choice due to that the scheme in [15] requires much computation and storage resource. Consequently, a specialized exploration on decision fusion based multi-slot SS is made in this paper. With theoretical analysis and simulation experiments, several significant conclusions are drawn as follows:

1. SS with one slot outperforms those with multiple slots if decision fusion rule is considered for multi-slot based SS.
2. Multi-slot based SS is conducive to the performance improvement of SS when instantaneous strong noise occurs in the radio environment.
3. For multi-slot based CSS, majority voting rule among multiple nodes obtains the optimal sensing performance.

The rest of this paper is organized as it is shown in the following. Section 2 reviews basic conceptions of SS and provide the system model. In Section 3, decision fusion-based SS in the multi-slot fashion is discussed in detailed. Simulations and analysis are made in Section 4. Finally, Section 5 concludes this paper.

## 2. System Model

Suppose that the frequency bands of interest are with carrier frequency $f_{c}$ and bandwidth W. Simultaneously, the received signal at the receiver of a CR is sampled at sampling frequency $f_{s}$ then the received signal y(n) could be formulated as the additive sum of clean signal (primary signal) s(n) and background noise w(n).

(1) y(n)=s(n)+w(n),n=0,1,…,N−1,

where N denotes the sampling point. If sensing duration is assumed to be T, $N=f_{s}\tau$, where τ denotes the sensing duration at a sensing event. In addition, s(n) is assumed to be independent and identically distributed random process with mean zero and variance $E\left[{\left|s\left(n\right)\right|}^{2}\right]=\sigma_{s}^{2}$ while w(n) is assumed to be additive white Gaussian noise (AGWN) with mean zero and variance $E\left[{\left|w\left[n\right]\right|}^{2}\right]=\sigma_{w}^{2}$. Take $\gamma =\frac{\sigma_{s}^{2}}{\sigma_{w}^{2}}$ as the received signal-to-noise radio (SNR) of the primary user. Note that the estimation of noise and signal variance could be made based on our work in [11].

For an ED, the energy statistics on average could be defined as:

(2) $$T\left(y\right)=\frac{1}{N}\sum_{n}^{N}{\left|y\left(n\right)\right|}^{2}.$$

When N is large enough, T(y) it is regarded to be Gaussian distribution. Consequently, the false alarm probability $P_{f}$ and its corresponding detection probability $P_{d}$ could be described as:

(3) $$P_{f}=P\left\{T\left(y\right)>\varepsilon |H_{0}\right\}=Q\left(\left(\frac{\varepsilon }{\sigma_{w}^{2}}-1\right)\sqrt{\tau f_{s}}\right),$$

(4) $$P_{d}=P\left\{T\left(y\right)>\varepsilon |H_{1}\right\}=Q\left(\left(\frac{\varepsilon }{\sigma_{w}^{2}}-\gamma -1\right)\sqrt{\frac{\tau f_{s}}{2\gamma +1}}\right),$$

(5) $$Q\left(x\right)=\frac{1}{\sqrt{2\pi }}\int_{x}^{\infty }exp\left(-\frac{t^{2}}{2}\right)dt,$$

where $H_{0}$ denotes the absence of the primary signal and $H_{1}$ denotes the presence of the primary signal. Additionally, Q(x) signifies the complementary cumulative distribution function (CCDF) of standard normal distribution function.

**Figure 1:**
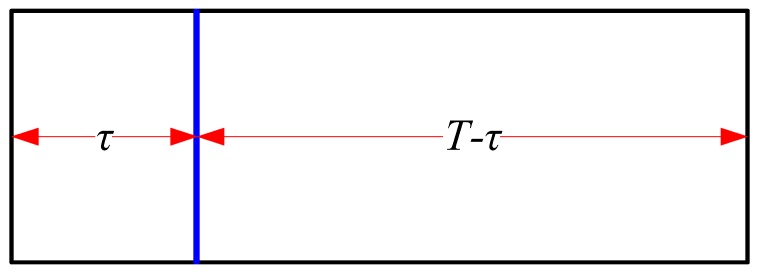
Frame structure of classical spectrum sensing (SS) schemes.

Figure 1 exhibits the frame structure of classical SS schemes, where T denotes the frame duration. For a fixed T, a larger τ corresponds to more sampling points and a better sensing performance in return. While a smaller τ corresponds to a larger T−τ and a better communication capacity of CR. In consequence, a tradeoff between sensing capacity and communication capacity always exists in CR based communication systems. How to take full advantage of limited sensing duration τ seems significant to balance sensing capacity and communication capacity of a CR.

## 3. Multi-Slot SS

**Figure 2:**
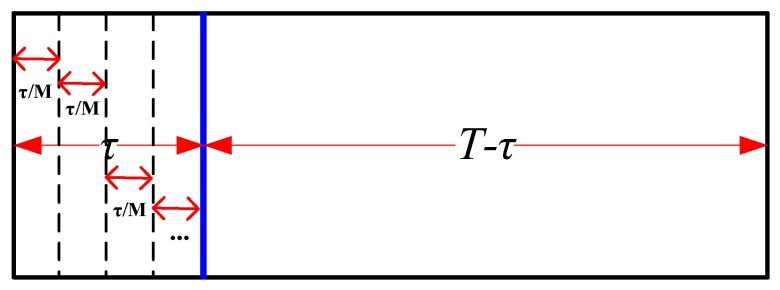
Frame structure of multi-slot SS.

In this section, the sensing slot in Figure 1 is divided into multiple discontinuous mini-slots just as shown in Figure 2, namely multi-slot SS. We give false alarm probability and detection probability of multi-slot SS based on decision fusion of mini-slots. Afterwards, we consider multi-slot SS in a cooperative fashion. In addition, we analyze the sensing performance of both local detection and cooperative detection when instantaneous strong noise occurs in the radio environment.

Figure 2 shows frame structure of multi-slot SS. Different from Figure 1, the sensing duration τ is decomposed into $M\;(M>0\&M\in Z_{+})$, where ED based SS is conducted based on joint decision of M mini EDs with decision fusion in the fusion center.

### 3.1. Multi-Slot SS with Single Node

Let M be the number of mini-slots and $\tau_{1}$ be the sensing time for each mini slot. Then the sensing duration in each frame could be denoted as $\tau \;=\;M\tau_{1}$, and the number of samples for each mini-slot is N1=NM. In order not to lose the generality, we assume that $N_{1}$ is an integer [16]. The observed signal at the receiver of each mini slot could be formulated as

(6) $$y_{i}\left(n\right)=h_{i}s_{i}\left(n\right)+w_{i}\left(n\right),n=0,1,\ldots ,N_{1},$$

where (6) is built under three assumptions: (1) $s_{i}\left(n\right)$ is independent and identically distributed random process with mean zero and variance $E\left[{\left|s_{i}\left(n\right)\right|}^{2}\right]=\sigma_{s}^{2}$ while $w_{i}\left(n\right)$ is AGWN with mean zero and variance $E\left[{\left|w_{i}\left[n\right]\right|}^{2}\right]=\sigma_{w}^{2}$; (2) $\left|h_{i}\right|$ follows the Nakagami−mi distribution with a mean of zero; (3) the noise of each slot is independent of each other and the spectrum detection of each slot is also mutually independent [17,18,19,20].

Denote $P_{f}^{\left(i\right)}$ and $P_{d}^{\left(i\right)}$ as the respective probabilities of false alarm and detection at the ith mini-slot. With detection threshold $\varepsilon_{0},$ the detection probability of ith time slot is formulated as:

(7) $$P_{d}^{\left(i\right)}=Q\left(\left(\frac{\varepsilon_{0}}{\sigma_{n}^{2}}-\gamma -1\right)\sqrt{\frac{\tau_{0}f_{s}}{2\gamma +1}}\right),$$

and the corresponding false alarm probability is described as:

(8) $$P_{f}^{\left(i\right)}=Q\left(\left(\frac{\varepsilon_{0}}{\sigma_{w}^{2}}-1\right)\sqrt{\tau_{0}f_{s}}\right).$$

For centralized CSS, the joint decision is made in the Fusion center (FC) with two patterns, data fusion and decision fusion. Data fusion is used to convey local received energy value of each mini EDs to FC and FC makes the final decision with fusion rules such as the maximum likelihood ratio (MLR), equal gain combining (EGC), selection combining (SC) and maximal ratio combining (MRC), etc. While logic-1 or legic-0 is transmitted to the FC for the final status decision of observed spectrum band in decision based CSS, where logic-1 denotes the presence of PU(primary user) and logic-0 denotes the absence of PU. It has been shown in [21,22,23,24] that soft-based decisions (decision fusion) outperform hard-based decisions (data fusion). But this difference in performance is at the expense of more energy consumption and bandwidth. Furthermore, [25] shows that the difference between hard and soft decision fusion is less than a fraction of 1 dB. However, in [26], a fair comparison shows that the hard decision fusion is better than that of the soft in most cases if we consider the reporting channel bandwidth. Motivated by these considerations, in this paper, we consider the decision fusion based CSS for multi-slot SS.

The K-out-of-N rule is a general rule for decision fusion-based CSS, where K denotes the number of CUs and states the existence of PU and N is the full number of CUs [27]. With a different size of K, several rules can be derived from the K-out-of-N rule, for instance the OR rule (K = 1) [28], the AND rule (K = N) [29] and majority-logic rule ($K\geq \left\lceil \frac{N}{2}\right\rceil$) [30]. In the following, we discuss three classical decision fusion based CSS mentioned above.

OR rule: The OR rule declares the presence of primary signal when at least one user indicates the signal is present. Mathematically, we define the status statistics in the FC as $\Lambda =\sum_{i=1}^{M}I_{i}$, where $I_{i}$ denotes the decision status of each local mini ED (0 or 1). If Λ≥1, we decide that the primary signal is present; otherwise, the primary user is not present. According to (7) and (8), the detection probability and false alarm probability in this case could be written as:

(9) $$P_{d-OR}=1-\prod_{i=1}^{M}\left(1-P_{d}^{\left(i\right)}\right),$$

(10) $$P_{f-OR}=1-\prod_{i=1}^{M}\left(1-P_{f}^{\left(i\right)}\right),$$

And rule: The primary signal is considered to be in when all the users declare its presence. In other words, if $\sum_{i=1}^{M}I_{i}=M$, the observed radio spectrum band is decided to be busy; in other cases, the observed radio spectrum band is idle. Then the corresponding detection probability and false alarm probability is denoted as:

(11) $$P_{d-AND}=\prod_{i=1}^{M}P_{d}^{\left(i\right)},$$

(12) $$P_{f-AND}=\prod_{i=1}^{M}P_{f}^{\left(i\right)},$$

Majority voting rule (MV): The majority voting rule declares the presence of the primary signal when more than half of CUs vote for that. Mathematically, if $\Lambda =\sum_{i=1}^{M}I_{i}\geq \left\lceil \frac{M}{2}\right\rceil$, the spectrums of interest are judged to be busy. On the contrary, the observed spectrums are unoccupied. As a result, the detection probability and false alarm probability could be expressed as

(13) $$P_{d-MV}=\sum_{j=0}^{M-\left[\frac{M}{2}\right]}\left(\begin{array}{c} M\\ \left[\frac{M}{2}\right]+j \end{array}\right){\left(1-P_{d,0}\right)}^{M-\left[\frac{M}{2}\right]-j}P_{d,0}^{\left[\frac{M}{2}\right]+j},$$

(14) $$P_{f-MV}=\sum_{j=0}^{M-\left[\frac{M}{2}\right]}\left(\begin{array}{c} M\\ \left[\frac{M}{2}\right]+j \end{array}\right){\left(1-P_{f,0}\right)}^{M-\left[\frac{M}{2}\right]-j}P_{f,0}^{\left[\frac{M}{2}\right]+j},$$

where $\left(\begin{array}{c} c\\ k \end{array}\right)=\frac{c!}{k!\left(c-k\right)!}$, $P_{d}^{\left(1\right)}=\cdots P_{d}^{\left(M\right)}=P_{d,0}\;$ and $P_{f}^{\left(1\right)}=\cdots P_{f}^{\left(M\right)}=P_{f,0}$.

**Proposition** **1.** *For a given false alarm probability, $\sigma_{w}^{2}$ and γ, $P_{d-OR}<P_{d}$ for a smaller M and $P_{d-OR}>P_{d}$ in large M cases.*

**Proposition** **2.** *For a given false alarm probability, $\sigma_{w}^{2}$ and γ, $P_{d-AND}\leq P_{d}$ ($P_{d-AND}\;=\;P_{d}$ when M=1).*

**Proposition** **3.** *For a given false alarm probability, $\sigma_{w}^{2}\;$ and γ, $P_{d-MV}<P_{d}$ with the rise of M.*

### 3.2. Multi-Slot Based CSS

In this subsection, multi-slot based SS is conducted in the cooperative fashion. We provide possible conditions of multi-slot based CSS and analyze their performance respectively [31]. 

**Figure 3:**
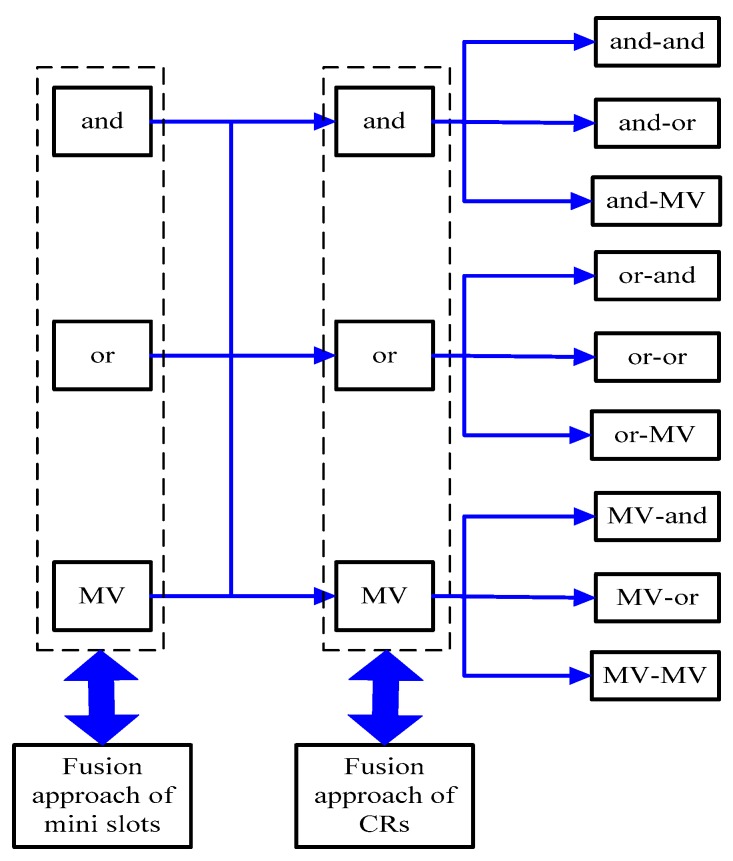
Possible conditions of multi-slot based cooperative spectrum sensing (CSS).

Figure 3 exhibits possible conditions of multi-slot based CSS, where fusion approaches of both mini slots and CRs consider three classical decision fusion rules mentioned in (9)–(14). It is emphasized that the ‘and’ fusion rule obtains a lower false alarm probability at the expense of a lower detection probability while the ‘or’ fusion rule gets a higher detection probability with a higher false alarm probability. As a result, neither the ‘and’ fusion rule nor the ‘or’ fusion rule are the optimal fusion rule for CSS. As for MV, a tradeoff is made therein between the capacity of protecting PU and the communication capacity of CUs. In consequence, in Figure 3, the sensing performance of the ‘and-and’ fusion rule based CSS, the ‘or-or’ fusion rule based CSS and the ‘MV-MV’ fusion rule based CSS are respectively similar to those of the ‘and’ fusion rule based CSS, the ‘or’ fusion rule based CSS and ‘MV’ fusion rule based CSS. In addition, the performance of the ‘and-or’ fusion rule based CSS and the ‘or-and’ fusion rule based CSS, the ‘and-MV’ fusion rule based CSS and the ‘MV-and’ fusion rule based CSS, the ‘or-MV’ fusion rule based CSS and the ‘MV-or’ fusion rule based CSS are approximate, theoretically, due to their symmetry of the fusion rule. On this basis analyzed above, we give the performance rank of nine fusion rules of multi-slot based CSS as exhibited in Table 1, where the rank is made given a tradeoff between detection probability and false alarm probability.

### 3.3. Instantaneous Strong Noise

Noise interference in the wireless communication environment greatly degrades communication quality and noise enhancement has been regarded as a hot issue on wireless communication in the long run [32]. When some emergency occurs in radio environment, such as thunder, lighting, moving objects, etc., the wireless signal will suffer from instantaneous strong interference, resulting in a dramatic decline on the quality of observed signal [33]. As a result, this paper assumes SS is conducted in instantaneous strong noise environment.

For SS with a single node, as instantaneous strong noise occurs, the practical energy statistics on average $T_{p}(y)$ will be larger than T(y) in (2),

(15) $$T_{p}(y)>T(y),$$

leading to that the practical false alarm probability $P_{f-p}$ and detection probability $P_{d-p}$ being above those in (3) and (4),

(16) $$\{\begin{array}{c} P_{f-p}>P_{f}\\ P_{d-p}>P_{d} \end{array}.$$

Note that (A11) is obtained based on two aspects of reasons. Firstly, the observed spectrums may be declared to be busy while it is idle in reality if instantaneous strong noise happens, which lowers the communication opportunity of CU. Secondly, the observed spectrums are busy originally and the detection probability and false alarm probability are higher than that of the actual when instantaneous strong noise occurs. This exaggerates the ability of CR to protect PU and lowers throughput of CU [34].

**Proposition** **4.** *For SS with single node, when $\varepsilon <(\sqrt{\frac{r}{2}+\frac{1}{4}}+\frac{1}{2})\sigma_{w}^{2}$, the disadvantages far outweigh advantages in instantaneous strong noise conditions.*

As a result, when sensing threshold $\varepsilon <(\sqrt{\frac{r}{2}+\frac{1}{4}}+\frac{1}{2})\sigma_{w}^{2}$, instantaneous strong noise will cause a performance decline on SS with single node. By dividing one time slot into multiple mini slots, the performance influence from instantaneous strong noise will be greatly restrained owing to joint decision of multiple slots at different moments.

**Proposition** **5.** *The sensing performance of multi-slot-based SS outperforms that of one-slot based SS when instantaneous strong noise occurs.*

Note that the proofs of Propositions 1–5 are respectively exhibited in Appendix A, Appendix B, Appendix C, Appendix D and Appendix E.

## 4. Simulations and Analysis

In this section, simulations are made to validate the conclusions drawn in this paper, where carrier frequency $f_{c}=100\;\mathrm{MHz}$, sampling frequency $f_{s}=300\;\mathrm{MHz}$, sampling number N=600 and SNR=−10 dB. Gauss channel, Rayleigh fading channel and Rice channel are considered in the simulations. 

### 4.1. Multi-Slot SS with Single Node

**Figure 4:**
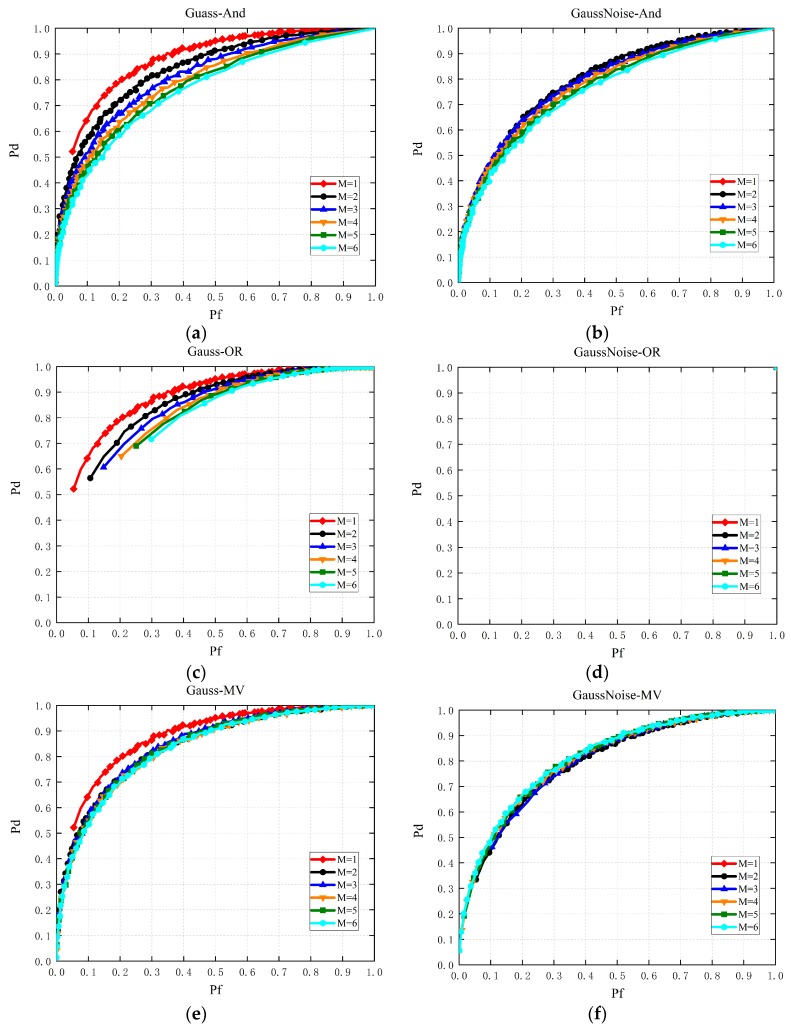
Performance comparisons of multi-slot SS in Gaussian channel with different fusion rules respectively ((**a**–**c**) are without instantaneous strong noise while (**d**–**f**) consider instantanous strong noise).

Figure 4 exhibits performance comparisons of multi-slot SS with single node in the Gaussian channel, where the major voting rule is short for major here and Figure 4d–f consider instantaneous strong noise in the simulations. Firstly, instantaneous strong noise has a prominent influence on sensing performance. From Figure 4a,d, when instantaneous strong noise occurs, the sensing performance greatly degrades. In addition, from Figure 4a–c, when M increases, the sensing performance decreases gradually, which manifests that SS with one slot outperforms those with multiple slots if decision fusion rule is considered for multi-slot based SS. Thirdly, from Figure 4e, when instantaneous strong noise occurs, the SS scheme with one slot doesn’t work due to the high false alarm probability. 

**Figure 5:**
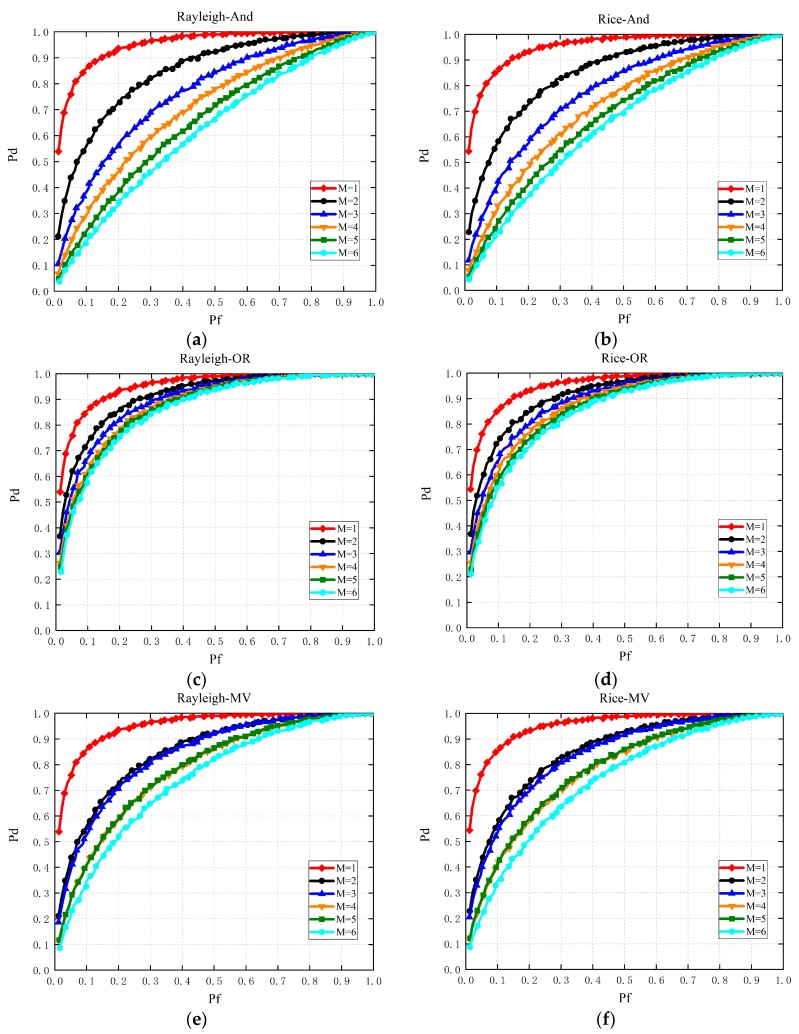
Performance comparisons of multi-slot SS in the Rice channel with different fusion rules for (**a**–**c**); performance comparisons of multi-slot SS in the Rayleigh channel with different fusion rules for (**d**–**f**).

As a supplement, Figure 5 shows performance comparisons of multi-slot SS with single node in the Rayleigh channel and the Rice channel, respectively. It validates that SS with one slot outperforms those with multiple slots if the decision fusion rule is considered for multi-slot-based SS again, just as analyzed in Propositions 1–3. In addition, when instantaneous strong noise occurs for one slot based SS, the detection capacity of the CR for idle radio spectrum decreases dramatically. This is because that the high false alarm probability makes it difficult to determine whether the primary signal exists. However, from Figure 4d,f, multi-slot SS could work normally, which validates that multi-slot based SS is conducive to the performance improvement of SS when instantaneous strong noise occurs, just as exhibited in Propositions 4–5.

### 4.2. Multi-Slot Based CSS

**Figure 6:**
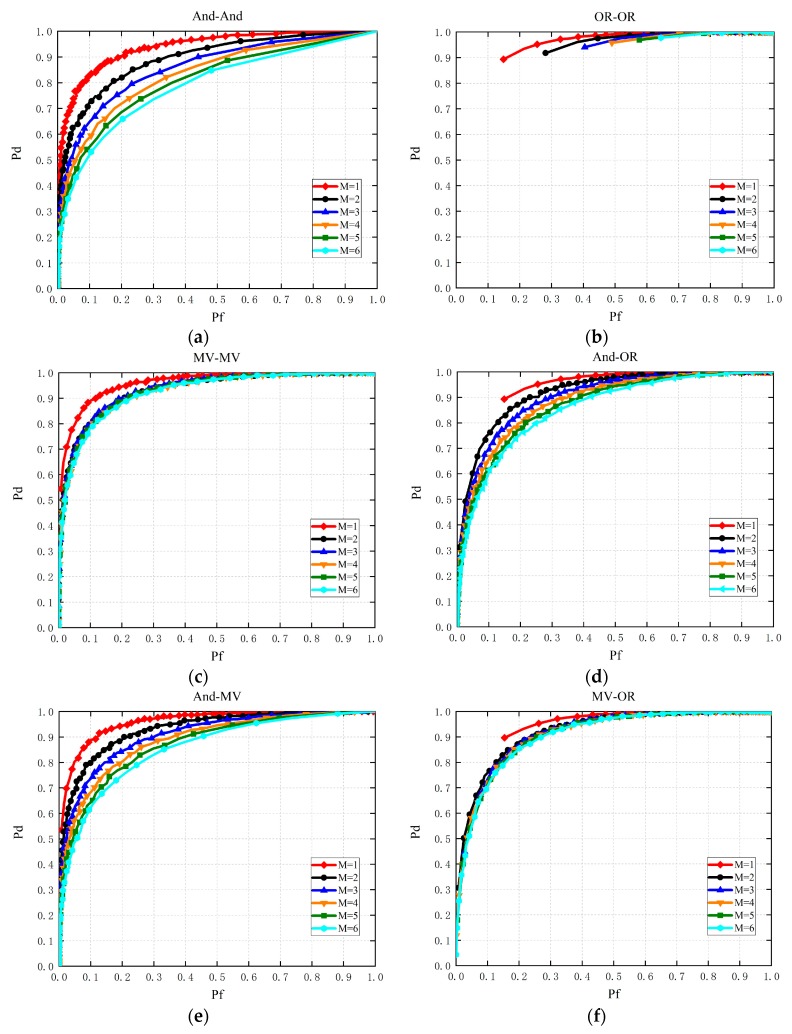
Performance comparisons of CSS with multiple slots in the Gaussian channel (**a**–**f**).

Performance comparisons of CSS with multiple slots in Gaussian channel is given in Figure 6. Obviously, the MV-MV rule-based CSS has the optimal sensing performance compared with the others. The OR-OR based sensing scheme almost does not work due to a higher false alarm probability, especially when M is at a higher level. The sensing performance of AND-AND based CSS is inferior to that of AND-OR, AND-MV and MV-OR, which corresponds with the discussions in Section 3.2. 

### 4.3. Performance Comparison with Data Fusion Based Multi-Slot SS

**Figure 7:**
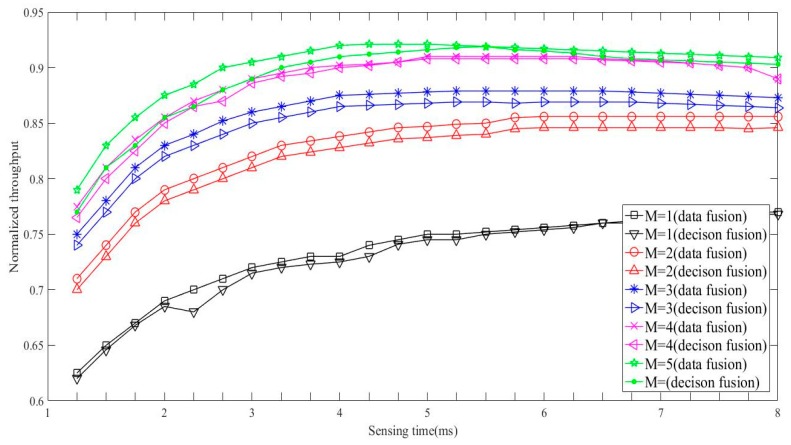
Throughput comparisons between the proposed scheme (decision fusion) and the classical scheme (data fusion).

**Figure 8:**
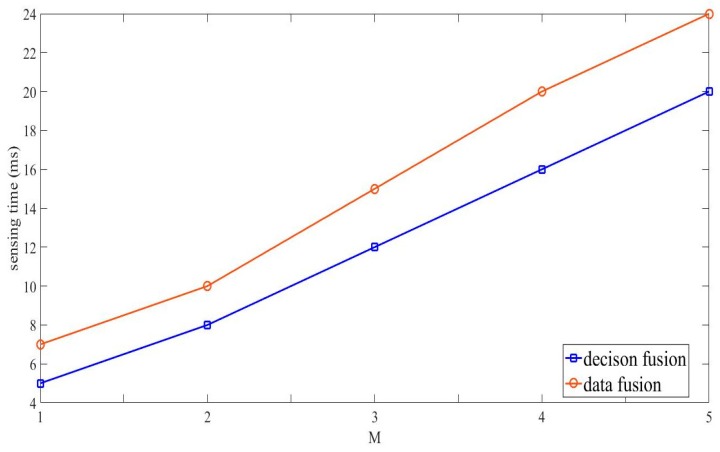
Sensing time comparions between the proposed scheme and the classical scheme.

Figure 7 and Figure 8 respectively exhibit normalized throughput and its corresponding sensing time comparisons between proposed scheme and classical scheme, where classical scheme denotes the work in [15] in the case that the fading coefficients are unknown, and the proposed scheme is with the MV fusion rule. Obviously, the classical scheme outperforms the proposed scheme in terms of sensing performance. However, the advantage of the classical scheme is at the expense of sensing time, which indicates that the classical scheme requires much more computation and storage resource than that of the proposed scheme. Therefore, the proposed scheme works as a better choice for the situation with limited storage and computation resource.

## 5. Conclusions

This paper is devoted to multi-slot based spectrum sensing with decision fusion rule and instantaneous strong noise, where several significant conclusions are drawn and demonstrated. On the basis of multi-slot based spectrum sensing with single node, we extend it to the cooperative fashion. Both theory analysis and simulation experiments validate the conclusions drawn in this paper. In the future work, convolutional neural network will be considered for possible performance improvement of multi-slot spectrum sensing.

## Figures and Tables

**Table 1:** Performance rank of nine fusion rules of multi-slot based CSS.

Performance Rank	Fusion Approach
1	MV-MV
2	and-MV, MV-and, or-MV, MV-or, and-or, or-and
3	and-and, or-or

## Appendix A

**Proof** **of** **Proposition** **1.** For given false alarm probability, $\sigma_{w}^{2}$ and γ, sensing threshold for SS and multi-slot SS with single node could be respectively formulated as

(A1) $$\varepsilon =[\frac{Q^{-1}(P_{f})}{\sqrt{N}}+1]\sigma_{w}^{2}$$

(A2) ε0=[Q−1(Pf)NM+1]σw2

Then

(A3) Pd−OR≥≤Pd⇔1−∏i=1M(1−Pd(i))≥≤Q((εσw2−γ−1)N2γ+1)⇔∏i=1MΦ((ε0σw2−γ−1)NM2γ+1)≥≤Φ((εσw2−γ−1)N2γ+1)

where Φ(⋅) = 1 − Q(⋅) denotes function of standard normal distribution. Due to

(A4) (ε0σw2−γ−1)NM2γ+1=[Q−1(Pf)NM−γ]NM2γ+1=Q−1(Pf)2γ+1−γNM2γ+1

(A5) $$\begin{array}{cc} \left(\frac{\varepsilon }{\sigma_{w}^{2}}-\gamma -1\right)\sqrt{\frac{N}{2\gamma +1}} & =[\frac{Q^{-1}(P_{f})}{\sqrt{N}}-\gamma ]\sqrt{\frac{N}{2\gamma +1}}\\ & =\frac{Q^{-1}(P_{f})}{\sqrt{2\gamma +1}}-\gamma \sqrt{\frac{N}{2\gamma +1}} \end{array}$$

therefore

(A6) (ε0σw2−γ−1)NM2γ+1≥(εσw2−γ−1)N2γ+1

On the other hand, Φ(x) monotonically increases on x. As a result,

(A7) Φ[(ε0σw2−γ−1)NM2γ+1]≥Φ[(εσw2−γ−1)N2γ+1]

(A7) is equivalent to

(A8) $$1-\left(1-P_{d}^{\left(i\right)}\right)\leq Q\left(\left(\frac{\varepsilon }{\sigma_{w}^{2}}-\gamma -1\right)\sqrt{\frac{N}{2\gamma +1}}\right)\Leftrightarrow P_{d-OR}<P_{d}$$

However, according to (19), ∏i=1MΦ((ε0σw2−γ−1)NM2γ+1)≤Φ((ε0σw2−γ−1)NM2γ+1)=1−(1−Pd(i)) due to Φ((ε0σw2−γ−1)NM2γ+1)∈[0,1]. In consequence, $P_{d-OR}$ gradually increases with the rise of M until $M_{0}$, after which $P_{d-OR}>P_{d}$. □

## Appendix B

**Proof** **of** **Proposition** **2.** According to (A8),

(A9) $$P_{d}^{\left(i\right)}\leq Q\left(\left(\frac{\varepsilon }{\sigma_{w}^{2}}-\gamma -1\right)\sqrt{\frac{N}{2\gamma +1}}\right)=P_{d}$$

Similarly, $P_{d}^{\left(i\right)}\in [0,1],$ then

(A10) $$P_{d-AND}=\prod_{i=1}^{M}P_{d}^{\left(i\right)}\leq Q\left(\left(\frac{\varepsilon }{\sigma_{w}^{2}}-\gamma -1\right)\sqrt{\frac{N}{2\gamma +1}}\right)=P_{d}$$

□

## Appendix C

**Proof** **of** **Proposition** **3.** According to Bernoulli experiment, if event A occurs with the probability p (0<p<1), then the probability that A happens *k* times in the *n*-weight Bernoulli experiment is equal to:

(A11) $$p_{n}(k)=\left(\begin{array}{c} n\\ k \end{array}\right)p^{k}{\left(1-p\right)}^{n-k}$$

For a given *n*, $\left(\begin{array}{c} n\\ k \end{array}\right)\;$ monotonously increases on k, $p^{k}$ and ${\left(1-p\right)}^{n-k}\;$ monotonously decreases with the rise of M. However, the increment of $\left(\begin{array}{c} n\\ k \end{array}\right)\;$ is unable to keep pace with the decline of $p^{k}{\left(1-p\right)}^{n-k}\;$ due to 0<p<1  and 0<1−p<1. As a result, $P_{d-MV}<P_{d}\;$ with the rise of M. □

## Appendix D

**Proof** **of** **Proposition** **4.** Let us respectively define the energy statistics in $H_{0}$ case and that in $H_{1}$ case as $T_{0}$ and $T_{1}$. Simultaneously, we assume the capacity of protecting PU is equal to its communication capacity for a CR. Then when instantaneous strong noise occurs,

(A12) $$\{\begin{array}{c} P_{f-p}=P\{T_{0}+\Delta >\varepsilon \}\\ P_{d-p}=P\{T_{1}+\Delta >\varepsilon \} \end{array}$$

where Δ denotes the average increment of energy statistics when instantaneous strong noise exits. (A12) is equivalent to

(A13) $$\{\begin{array}{c} P_{f-p}=P\{T_{0}>\varepsilon_{1}\}\\ P_{d-p}=P\{T_{1}>\varepsilon_{1}\} \end{array}$$

due to an increase on both false alarm probability and detection probability, where $\varepsilon_{1}\;(0<\varepsilon_{1}<\varepsilon )$ denotes a lower sensing threshold. The increment of false alarm probability and detection probability when instantaneous strong noise happens are respectively defined as:

(A14) $$\begin{array}{ll} \Delta P_{f} & =P_{f-p}-P_{f}\\ & =P\{T_{0}>\varepsilon_{1}\}-P\{T_{0}>\varepsilon \}\\ & =Q\left(\left(\frac{\varepsilon_{1}}{\sigma_{w}^{2}}-1\right)\sqrt{\tau f_{s}}\right)-Q\left(\left(\frac{\varepsilon }{\sigma_{w}^{2}}-1\right)\sqrt{\tau f_{s}}\right) \end{array}$$

(A15) $$\begin{array}{ll} \Delta P_{d} & =P_{d-p}-P_{d}\\ & =P\{T_{1}>\varepsilon_{1}\}-P\{T_{1}>\varepsilon \}\\ & =Q\left(\left(\frac{\varepsilon_{1}}{\sigma_{w}^{2}}-\gamma -1\right)\sqrt{\frac{\tau f_{s}}{2\gamma +1}}\right)-Q\left(\left(\frac{\varepsilon }{\sigma_{w}^{2}}-\gamma -1\right)\sqrt{\frac{\tau f_{s}}{2\gamma +1}}\right) \end{array}$$

For

(A16) $$\frac{\partial P_{f}}{\partial \varepsilon }=-\frac{\sqrt{\tau f_{s}}}{\sqrt{2\pi }\sigma_{w}^{2}}\exp\{-\frac{1}{2}{\left[(\frac{\varepsilon }{\sigma_{w}^{2}}-1)\sqrt{\tau f_{s}}\right]}^{2}\}$$

(A17) $$\frac{\partial P_{d}}{\partial \varepsilon }=-\frac{\sqrt{\tau f_{s}}}{\sqrt{2\pi (2\gamma +1)}\sigma_{w}^{2}}\exp\{-\frac{1}{2}{[(\frac{\varepsilon }{\sigma_{w}^{2}}-\gamma -1)\sqrt{\frac{\tau f_{s}}{2\gamma +1}}]}^{2}\}$$

(A18) $$\frac{\sqrt{\tau f_{s}}}{\sqrt{2\pi }\sigma_{w}^{2}}>\frac{\sqrt{\tau f_{s}}}{\sqrt{2\pi (2\gamma +1)}\sigma_{w}^{2}}$$

then

(A19) $$\begin{array}{ll} \frac{\partial P_{f}}{\partial \varepsilon }\begin{array}{c} \geq \\ \leq \end{array}\frac{\partial P_{d}}{\partial \varepsilon } & \Leftrightarrow {\left[(\frac{\varepsilon }{\sigma_{w}^{2}}-1)\sqrt{\tau f_{s}}\right]}^{2}\begin{array}{c} \geq \\ \leq \end{array}{[(\frac{\varepsilon }{\sigma_{w}^{2}}-\gamma -1)\sqrt{\frac{\tau f_{s}}{2\gamma +1}}]}^{2}\\ & \Leftrightarrow (2\gamma +1){\left(\frac{\varepsilon }{\sigma_{w}^{2}}-1\right)}^{2}\begin{array}{c} \geq \\ \leq \end{array}{(\frac{\varepsilon }{\sigma_{w}^{2}}-\gamma -1)}^{2}\\ & \Leftrightarrow {\left(\frac{\varepsilon }{\sigma_{w}^{2}}-\frac{1}{2}\right)}^{2}\begin{array}{c} \geq \\ \leq \end{array}\frac{\gamma }{2}+\frac{1}{4}\\ & \Leftrightarrow \varepsilon \begin{array}{c} \geq \\ \leq \end{array}(\sqrt{\frac{r}{2}+\frac{1}{4}}+\frac{1}{2})\sigma_{w}^{2} \end{array}$$

According to A(16)–(19), when $\varepsilon <(\sqrt{\frac{r}{2}+\frac{1}{4}}+\frac{1}{2})\sigma_{w}^{2}$, $\left|\frac{\partial P_{f}}{\partial \varepsilon }\right|>\left|\frac{\partial P_{d}}{\partial \varepsilon }\right|$, which indicates that the increment of false alarm probability is higher than that of detection probability when $\varepsilon <(\sqrt{\frac{r}{2}+\frac{1}{4}}+\frac{1}{2})\sigma_{w}^{2}$. Due to the fact that false alarm probability is against of sensing performance while detection probability is to the benefit of sensing performance, consequently, when $\varepsilon <(\sqrt{\frac{r}{2}+\frac{1}{4}}+\frac{1}{2})\sigma_{w}^{2}$, disadvantages far outweigh its advantages in instantaneous strong noise conditions for SS with single node. □

## Appendix E

**Proof** **of** **Proposition** **5.** According to (15) and (16), when instantaneous strong noise occurs, the detection probability and its corresponding false alarm probability will be dramatically improved for one-slot based SS. However, high false alarm probability and detection probability correspond to an increased difficulty in distinguish between the signal from PU and environment noise, which implies a great decline on sensing performance and even a failure in detecting observed spectrum bands. For multi-slot based SS, the influence of instantaneous strong noise is balanced due to multiple time slots, especially when the time slot is large enough. In consequence, the sensing performance of multi-slot based SS has a slight decline and even remains unchanged. To sum up, the sensing performance of multi-slot based SS outperforms that of one-slot based SS when instantaneous strong noise occurs. □
